# MENGA: A New Comprehensive Tool for the Integration of Neuroimaging Data and the Allen Human Brain Transcriptome Atlas

**DOI:** 10.1371/journal.pone.0148744

**Published:** 2016-02-16

**Authors:** Gaia Rizzo, Mattia Veronese, Paul Expert, Federico E. Turkheimer, Alessandra Bertoldo

**Affiliations:** 1 Department of Information Engineering, University of Padova, Padova, Italy; 2 Department of Neuroimaging, Institute of Psychiatry, Psychology & Neuroscience, King’s College London, London, United Kingdom; University of Texas at Austin, UNITED STATES

## Abstract

**Introduction:**

Brain-wide mRNA mappings offer a great potential for neuroscience research as they can provide information about system proteomics. In a previous work we have correlated mRNA maps with the binding patterns of radioligands targeting specific molecular systems and imaged with positron emission tomography (PET) in unrelated control groups. This approach is potentially applicable to any imaging modality as long as an efficient procedure of imaging-genomic matching is provided. In the original work we considered mRNA brain maps of the whole human genome derived from the Allen human brain database (ABA) and we performed the analysis with a specific region-based segmentation with a resolution that was limited by the PET data parcellation. There we identified the need for a platform for imaging-genomic integration that should be usable with any imaging modalities and fully exploit the high resolution mapping of ABA dataset.

**Aim:**

In this work we present MENGA (Multimodal Environment for Neuroimaging and Genomic Analysis), a software platform that allows the investigation of the correlation patterns between neuroimaging data of any sort (both functional and structural) with mRNA gene expression profiles derived from the ABA database at high resolution.

**Results:**

We applied MENGA to six different imaging datasets from three modalities (PET, single photon emission tomography and magnetic resonance imaging) targeting the dopamine and serotonin receptor systems and the myelin molecular structure. We further investigated imaging-genomic correlations in the case of mismatch between selected proteins and imaging targets.

## Introduction

Transcriptome-wide mappings (such as the microarray-based Allen Human Brain Atlas, ABA [1]) provide information on gene expression patterns and consequently about system proteomics. They offer a great potential for neuroscience research since they can be used as reference standards to establish validity of modern *in vivo* imaging technologies. For example, these patterns have been correlated with the binding patterns of radioligands targeting specific molecular systems and imaged with positron emission tomography (PET) in unrelated control groups [2]. One can easily see how a similar approach can be extended to other functional and structural modalities (single photon emission tomography SPET, magnetic resonance imaging MRI).

In all instances, the mRNA platform could also be used to compare the accuracy of competing numerical methodologies for the quantification of the imaging data; for example one could use the genomic reference to aid the selection of the optimal kinetic modeling approach for PET data [2–4] or to infer about the sensitivity of a given imaging modality to its molecular target [5]. This is potentially applicable to any imaging modality, although an efficient procedure of imaging-genomic matching is required.

The approach however cannot be easily generalized as post-transcriptional events and translational mechanisms may regulate the protein expression of specific genes hence disrupting the tight correlation between transcripts and proteins [6]. Inconsistent correlations between mRNA and protein concentrations have been reported in the literature [7], and these may not be mutually exclusive. First, there are many complex and varied post-transcriptional mechanisms that are involved in turning mRNA into protein (e.g. transcriptional and post-transcriptional splicing, translational modifications and protein complex formations); second, proteins may differ substantially in their *in vivo* half-lives; and/or third, technologies may not be perfectly accurate in measuring either mRNA or protein content. To these caveats, one has to add the limitations of post-mortem tissue availability and quality that may further hamper the predictive power of genomic brain maps to the *in vivo* protein cohorts.

Indeed, the integration of brain mRNA atlases with neuroimaging might help to address the inverse problem: MENGA offers a platform to compare gene-expression vs. protein-expression as provided by an imaging modality that returns a reliable measure of *in vivo* protein expression so that one can select the mRNA probe with highest predictive value for protein content [8]. Clearly, one has to consider whether to use mRNA or image profiles as benchmark in the study. The choice must depend on the purpose of the experiment and on what measure can be considered a priori more reliable.

We were the first to attempt the integration of mRNA and PET data using a region-based segmentation [2]; in the original methodology however we did not take advantage of the high resolution level of the genomic sampling of the ABA database and our analyses focused solely on PET data as unique imaging modality. Other recent alternatives to integrate ABA data and imaging are region-based and therefore do not use the full ABA database being based on a specific anatomical atlas [9], contrary to MENGA, which is atlas naive. Hence we identified the need for a platform for imaging-genomic integration, which should: a) be usable with any imaging modalities and b) employ a segmentation method that exploits the full potential of ABA dataset and its anatomical labeling.

In the present work we present MENGA (Multimodal Environment for Neuroimaging and Genomic Analysis) that provides an optimal computational environment to investigate the correlation patterns between neuroimaging data (both functional and structural) with ABA mRNA gene expression profiles. MENGA uses the complete ABA database with its metadata and anatomical segmentation.

To assess the face validity of the method we applied MENGA to six different imaging datasets that target the dopamine and serotonin receptor systems and the myelin molecular structure in the human brain. The analyses included PET, SPET, MRI data. We further investigated the specificity of the approach in the case of mismatch between selected proteins and imaging targets.

## The MENGA Platform

This section describes MENGA as software package, presenting its features, functionalities and functioning hierarchy.

### Software description

MENGA is a comprehensive license-free tool (only for academic use) for the integration of brain imaging modalities and Allen human brain genomic atlas. The latest released version is available at http://fair.dei.unipd.it/software/, at the NITRC website (http://www.nitrc.org/projects/menga/) and at GitHub (https://github.com/FAIR-CNS/MENGA). MENGA has been developed in a Matlab® environment (The Mathworks Inc., Natick, MA, USA–Matlab 2012a version and above) and consists of a group of Matlab functions together with an in-house processed version of the ABA database (in Appendix).

**Fig 1:**
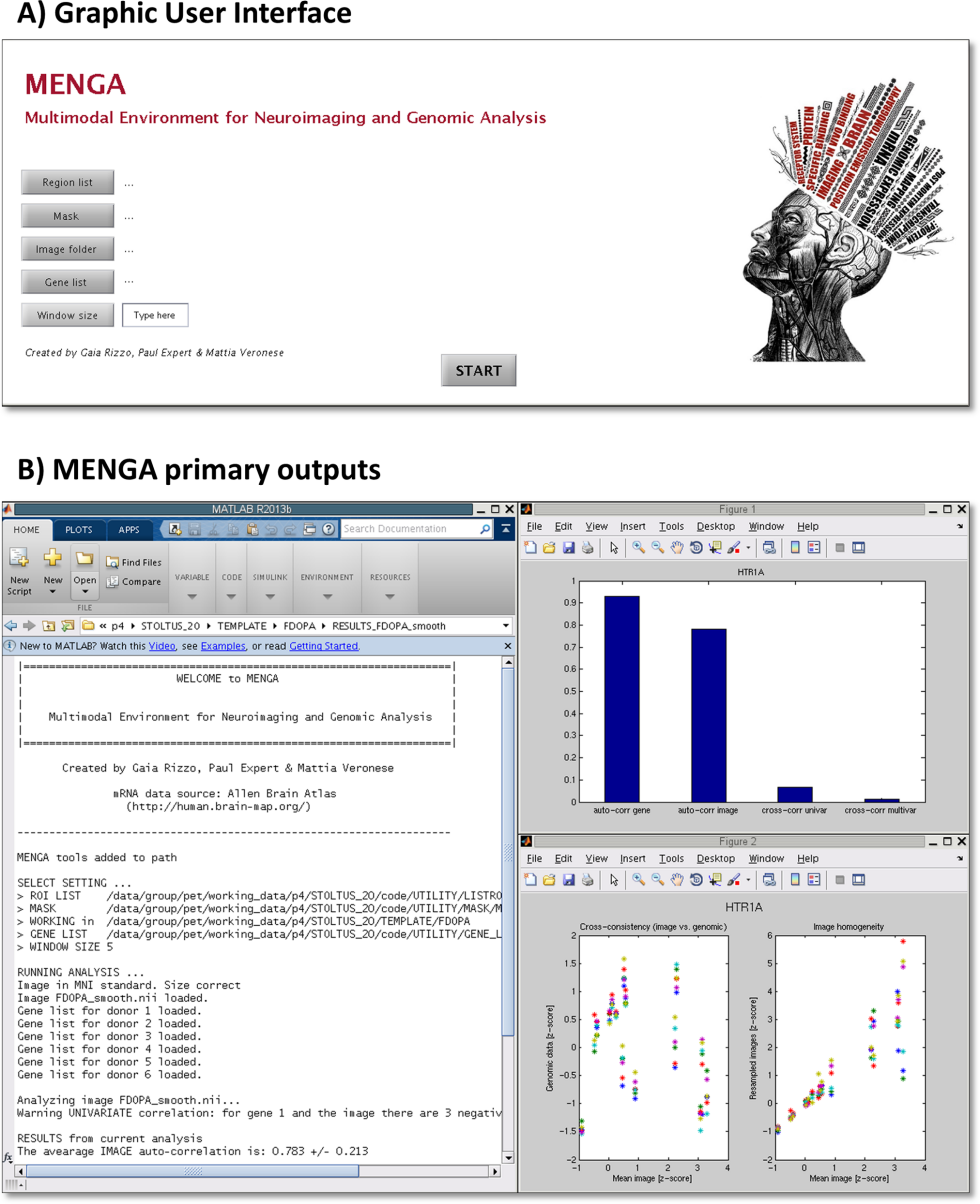
MENGA software. A) Matlab-based graphic user interface. B) Overview of MENGA’s outputs. Left: Printed result report on Matlab workspace. Right: summary statistics and scatter analysis plot for gene vs. image cross-correlation and image auto-correlation.

MENGA works with a graphical user interface (GUI) which makes it usable by both experts and IT naïve users (Fig 1). The only system requirement for MENGA is ~7 GB of disk usage that is necessary to store the processed copy of the ABA database.

### Workflow

**Fig 2:**
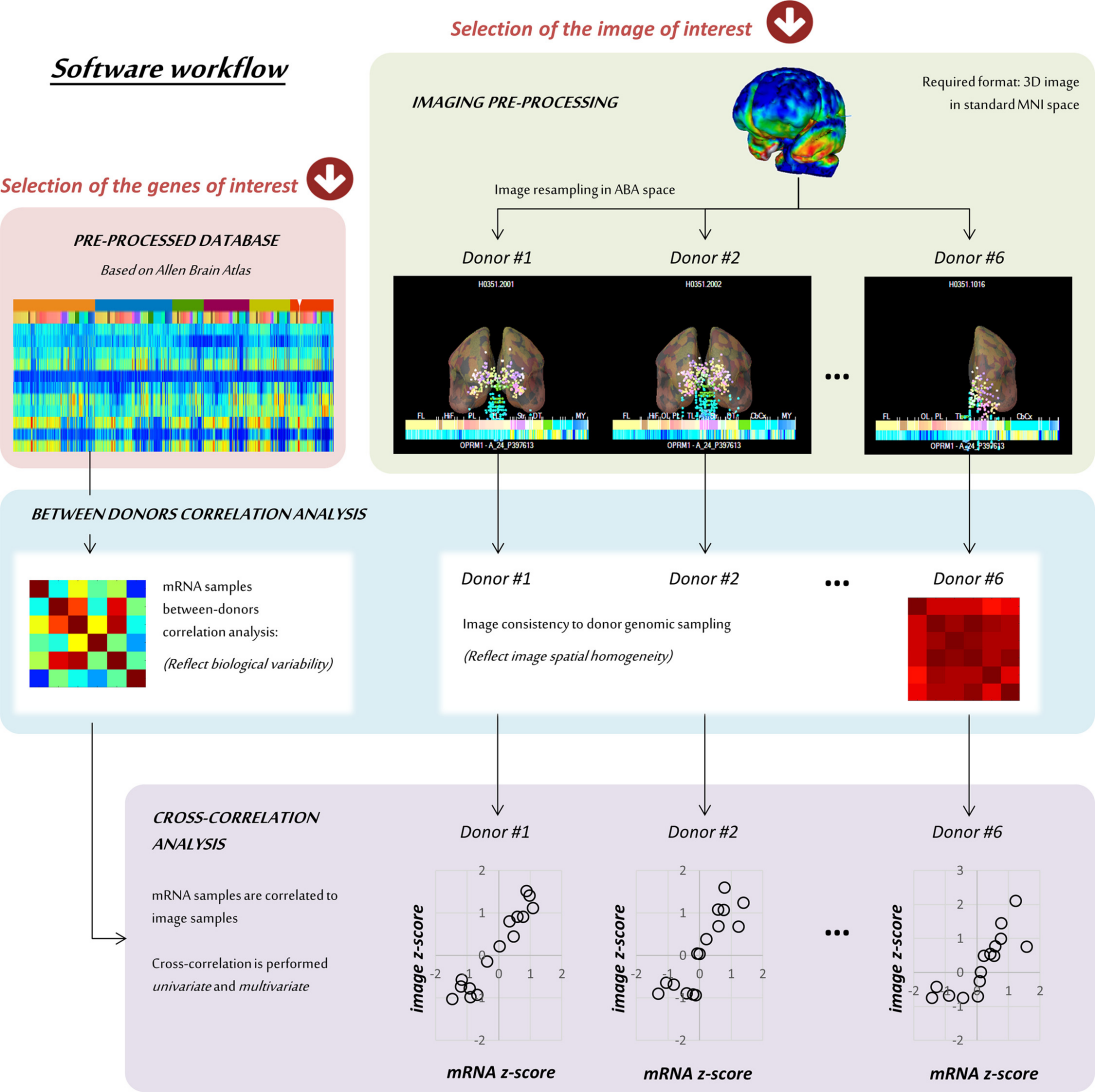
MENGA workflow and functional blocks. MENGA requires as input an image to process and a list of genes of interest to compare with. The image needs to be a 3D matrix already in standard MNI format (1mm, 181x217x181 or 182x217x182). The genes are selected from a pre-processed version of the Allen human brain database, where each protein is identified with a unique transcript profile. Once the image is resampled into the mRNA donor spaces, MENGA performs between-subject correlation analysis for both mRNA and image data. As last step, the cross-correlation analysis between mRNA and imaging is calculated for all the selected genes.

The software workflow is presented in Fig 2. The analysis starts with the user selecting the images to be processed and the list of genes of interest for the comparison.

The mRNA data of the donors are loaded from the MENGA database. Details of the processing and generation of this database can be found in the Appendix. The images are resampled in ABA space, determining for each genomic sample a corresponding image sample calculated within a window of user-defined size. The user has the choice to select an image mask, i.e. a binary 3D image to limit the analysis in the areas of interest removing the background. All the analyses are then carried by combining groups of single samples based on their anatomical labeling, with the level of resolution defined by the user.

Once the image matching to the database is completed, MENGA performs three different types of correlations: 1) the between-donors correlation calculated on mRNA (hereafter referred to as gene *auto-correlation*), 2) the between-donors correlation calculated on image data (hereafter referred to as image *auto-correlation*) and 3) the correlation analysis for each gene of the list with the image samples (hereafter referred to as *cross-correlation*).

Auto-correlation is calculated as Pearson’s linear correlation, with the genomic auto-correlation returning information about the biological variability of the mRNA expression and the image auto-correlation measuring the image homogeneity to the different donor tissue sampling. In fact, all the 6 donors have been sampled in the same anatomical regions, but each region is described by different samples across donors, both in number and position. This implies that the same image sampled in different donor’s space might have different regional values due to heterogeneity of anatomical sampling and heterogeneity and noise of mRNA measurements. The genomic auto-correlation aims to give a measure of this heterogeneity and noise. The cross-correlation represents instead the main output of the software analysis, where the genomic data are correlated to the image values, both in a *univariate* and *multivariate* manner.

### Logical blocks

#### mRNA pre-processing

This block aims to define for each donor a unique mRNA expression profile for each gene of the ABA merging the genomic samples at the level of resolution selected by the user.

MENGA queries the genomic data from a processed version of the ABA database included in the software package (see Appendix for details on the generation of this database).

The mRNA data and sample metadata necessary for the analysis are based on the list of genes selected by the user and aggregated in regions of interest (ROIs) identical for all ABA donors. For each ROI, a single mRNA value is hence calculated from the average of the samples within the region. The variability of the sample expression within each ROI as well as the number of regional samples are also saved and used to define correlation weights. We implemented a weighted correlation for the image-genomic cross-correlation to take into account the variability of the measurements, in order to weigh more the most reliable regional values, i.e. values obtained from the average of a higher number of samples in the region and with smaller variability in the region. Further details on the weights definition can be found in the “Image and mRNA cross-correlation” section. We point out that as the number and spatial locations of genomic samples varies across donors it is impossible to perform any correlation analysis at *sample level*. Nevertheless, different levels of resolution are available: single brain structures or coarse regions, depending on the user’s interest.

#### Image pre-processing

This block aims to resample the image from MNI space that is the standard reference space for neuroimages to ABA coordinates in order to obtain a 1:1 image-to-sample correspondence for each donor of the dataset and sample the data at the level of resolution selected by the user. This resampling is performed on every image considering the ABA coordinates of each donor separately since the tissue sampling for the microarray analysis has not been performed in the exact same coordinate system for all the subjects.

The first step consists into importing all the images into the Matlab environment. Each image is analyzed separately. The images must be 3D, already in MNI space and oriented according to the neurological convention. MENGA accepts images both in MNI ICBM152 coordinates (image size: 181x217x181 voxels) [10] and FSL MNI coordinates (image size: 182x218x182) and converts them into the MNI ICBM152 convention (voxel size 1mm x 1mm x 1mm).

The image intensity data are then normalized to z-scores (subtracting the global mean as the centering factor and dividing by the standard deviation as the normalization factor) and masked to remove background pixels. The mask can also be created to limit the analysis to certain regions or slices of interest. The voxels outside the mask are set to “not-a-number” (NaN) and then discarded from further analysis. Mask definition is user-dependent and is also selected through the GUI.

After masking, the images are resampled in the genomic space independently for each donor of the ABA database and an image sample is defined for each genomic sample available. Spatial matching is based on MNI coordinates as provided by ABA. Each image sample is calculated as the average of the voxels within a 3D window centered around the MNI coordinates of the genomic sample. Users can select the size of the window through the GUI. This should reflect the effective spatial resolution of the image being analysed. If the window includes voxels outside the mask, the sample is discarded. This conservative approach allows all the image samples to be obtained from the same number of voxels.

As the last step of the processing, the image samples are aggregated in the regions defined by the user, following the same procedure of the mRNA pre-processing described in the previous section. Therefore for each single image to be analyzed we obtain six re-mapped images.

#### Image and mRNA auto-correlation analysis

This block computes between-donors autocorrelation for both imaging and genomic data at the pre-selected resolution level. The analysis is done by calculating the squared Pearson’s correlation coefficient (*R*^2^) of the mRNA/image data for each pairs of donors returning a symmetric matrix of 6x6 elements for each image/gene considered. The summary statistics (as mean and standard deviation across the different couplings) are displayed on the Matlab workspace and stored in the report file.

#### Image and mRNA cross-correlation

In the analysis step, MENGA performs the cross-correlation between mRNA and image data in two ways, univariate and multivariate.

The univariate cross-correlation analysis consists in the weighted regression of mRNA and image data for each donor. The weights are defined as the ratio of the number of samples in each region over the variability of the image data in that region for each subject. Specifically, the higher the number of samples the smaller is the expected variability in a ROI. The resulting squared Pearson’s correlation coefficients (*R*^2^) are displayed in a matrix of 6x1 elements and are reported in the summary statistics as mean and standard deviation across the different donors. Note that MENGA does not return the significance of the correlation coefficient *R*^2^ which can be derived by the user from the value of the coefficient itself and the number of regions used in the correlation. For the simplified coarse region list (i.e. 15 ROIs) the correlation is significant for a *R*^2^ > 0.25 (p-value<0.05).

MENGA returns also information on the directionality of the correlation as additional output of the analysis, as the number of times (out of 6 matches) for which MENGA finds a direct (positive) correlation. This value is reported in the command window during the analysis and stored in the summary report.

The multivariate cross-correlation consists of a weighted multiple regression between the mRNA data of the different donors (corresponding to the independent variables) and the mean of image regional values across donors (corresponding to the dependent variable). This is achieved by solving the following model:

$$y=b_{0}+b_{1}x_{1}+b_{2}x_{2}+\cdots +b_{k}x_{k}$$ (1)

where *y* refers to the average of the image values re-mapped in the donors space; *x*_1_, …, *x*_*k*_ are the predictors (the donor mRNA data), *b*_0_ the intercept, and *b*_1_ …, *b*_*k*_ the regression coefficients. The model fitting performance is defined by the adjusted coefficient of determination (or multivariate correlation coefficient) ($R_{adj}^{2}$) representing the amount of the total image variability explained by the genomic data. The adjusted *R*^2^ is defined as:

$$R_{adj}^{2}=1-(1-R^{2})\;\left[\frac{n-1}{n-(k+1)}\right]$$ (2)

where *n* is the sample size and *k* is total number of explanatory variables in the model (not including the constant term). The adjusted *R*^2^ can be negative and is always smaller than or equal to that of *R*^2^.

Like the correlation coefficient for simple linear regression, this index returns information about the genomic-imaging cross-correlation level. Since it is expected that the 6 donors should carry similar information in terms of gene expression (modulo the biological variability of the process), there is the possibility of overfitting the regression model and overestimating the coefficient of determination by using too many predictors that are statistically non significant. In addition, literature guidelines suggest that no more than *n*/10 predictors should be included at a time [11]. For these reasons MENGA first applies a principal component analysis (PCA) to the mRNA of the 6 donors in order to extract the principal components that account for at least 95% of the total variance in the data, and then performs the regression analysis. Notably, depending on the between-donors variability of the protein expression under study, the number of selected components varies, usually between 1 and 3. As second step, a weighted regression of the image data is performed using the selected principal components as regressors. The weights are defined as the ratio of the average of the number of samples in each region across the re-mapped subjects and the between-subject variability of the image values in the same region. As within the univariate analysis, a larger number of samples in a region leads to a smaller variability across mappings (i.e. the highest the image homogeneity), and a more reliable regional value.

The multilinear analysis is completed with the calculation of the chance likelihood. The chance likelihood represents a measure of cross-correlation reliability, as it returns the probability that the predictors (genomic data) are unrelated to the outcome (the image values): the smaller the likelihood, the smaller the probability that the estimated *R*^2^ is due to chance.

This value is obtained via a bootstrapping approach, resampling 1,000 times the genomic data and re-calculating the coefficient of determination. The chance likelihood is calculated as:

$$chance\;likelihood=\frac{number\;of\;instance(R^{2}>R_{or}^{2})}{number\;of\;bootstraps}$$ (3)

where $R_{or}^{2}$ is the value of the coefficient of determination obtained from the real data and *R*^2^ is the value obtained using the bootstrapped genomic data.

We want to highlight that the statistical methods implemented in MENGA are parametric and can have therefore some limits (such as assuming linearity and normal distribution). Therefore, the user must be careful with the linearity assumptions of these statistical approaches. However, one main outcome of the software is the matching of the gene and image information in the same space, which are then stored in the results files and in the summary. Once the analysis is complete, the user can easily retrieve these data and apply any more advanced non-parametric statistics suitable for his/her needs.

Also in the case of the multivariate analysis, MENGA returns information on the directionality of the correlation with respect to the first principal component (as direct or positive (+) and negative (-) correlation). This information is reported in the command window and stored in the summary report. In summary, the final results of the pipeline are the average genomic and image auto-correlation across donors, the average univariate cross-correlation and the multivariate cross-correlation.

### Input & output data management

Input data are managed by using text files (.txt) for the gene selection and NIfTI (.nii) or Analyze format (.hdr/.img) for the images. For *region selection* and the *image masking*, MENGA includes a set of libraries of possible lists of regions of interest and MNI brain masks. The user can choose from the already implemented solutions or extend the libraries him/herself. In particular, MENGA already includes several default lists of regions, derived from the *structure* and *coarse* level defined in the ABA. The ABA coarse level lists 26 ROIs although some of them are under sampled (less than 2 samples) or segmented at a level of resolution inconsistent with the others. For this reason, we also included in MENGA a simplified version of the coarse level, with 15 ROIs of 38±32 (mean±SD) samples each (see S1 Table). The ABA *structure* level of interest is also included in MENGA for completeness, but its use could be non trivial for the interpretation and localization of the regions of interest. The user can also expand the library of ROI lists, defining a new list of regions. This list must contain the ABA labels as presented in the Allen website and each region of interest can be defined as a combination of samples, structures or coarse regions (for an ontology of the regions of interest, please refer to the ABA website or to the software manual).

MENGA comes with a series of default options: the default region list corresponds to the *simplified coarse* level of resolution [1] and the default mask is the binary image derived from the FSL MNI ICBM152 template [10], limited to the left hemisphere, to benefit from the contribution of all donors. The default value of the window size is 5 mm, set to match the resolution level of imaging techniques with discrete spatial resolution such as PET. Defaults can be modified depending on the particular needs of the user.

After the analysis a report with all the correlation statistics is displayed in the Matlab workspace and saved as text file (.txt) in the working folder. The text file contains also the setting information used for the analysis and a copy of the raw genomic data (in z-score). Additionally, MENGA generates two figures (each one saved as both.jpg and.fig files) summarizing genomic and image correlation statistics and the corresponding scatter plots (Fig 1B).

A Matlab file for expert users is also stored in the result directory recording all of the internal results. Further details about MENGA input requirements and software outputs are described in the user manual available with the MENGA software package.

## Application to Case Studies

To evaluate the program performances, MENGA was tested on a representative set of image data acquired with different modalities (PET, MRI and SPET). The analysis was performed considering both matched and miss-matched genomic information. The following subsections summarize the main features of the datasets available and the results obtained.

### Image dataset

We focused our analysis on the serotonin and the dopamine systems as well as myelin in brain tissue. In particular, we analyzed the serotonin system with the [^11^C]CUMI (from here on, [^11^C]CUMI indicates [^11^C]CUMI-101) and [^11^C]WAY100635 PET tracers, which target the 5HT1A receptors (as agonist and antagonist respectively) [2]. For each tracer, we created a template by averaging healthy controls protein expression density maps.

Pre and post-synaptic proteins of the dopamine pathway were analyzed with templates of PET and SPET images. [^18^F]FDOPA PET and [^123^I]FP-CIT SPET templates were used as markers of the dopamine synthesis capacity (DDC, dopa decarboxylase enzymatic rate) and the dopamine transporter (DAT) respectively. The templates are freely available at the NITRC website (http://www.nitrc.org/projects/spmtemplates/) [12]. A template for [^11^C]Raclopride PET data was obtained by averaging healthy subjects data that have been previously published [13] and used as D_2_/D_3_ receptor density marker.

**Fig 3:**
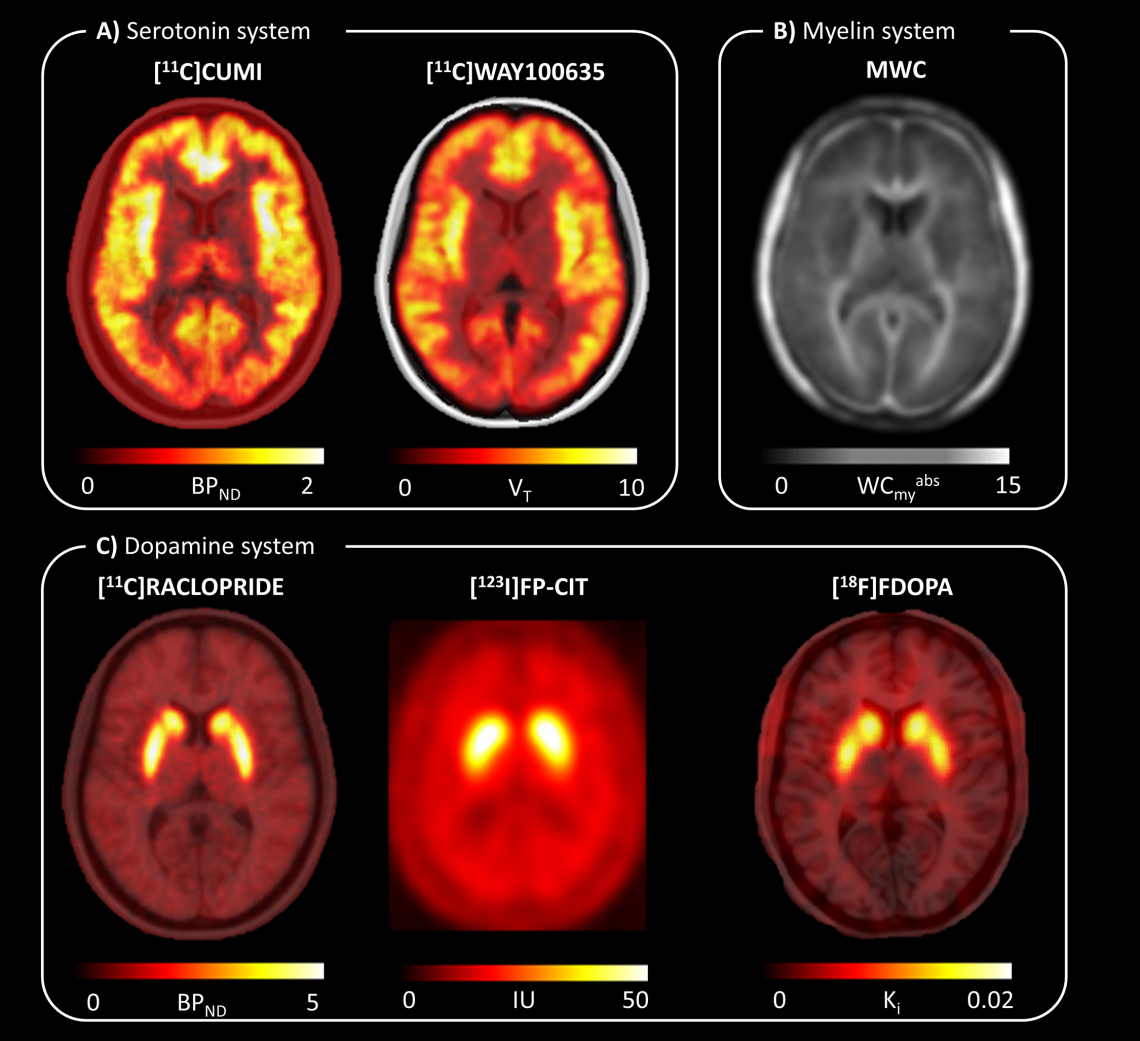
Tested neuroimaging modalities. A) Serotonin system—[^11^C]CUMI and [^11^C]WAY100635 PET imaging are used as 5HT_1A_ receptor markers (agonist and antagonist respectively). The protein density maps are images of non-displaceable specific binding, *BP*_*ND*_ [unitless] and volume of distribution, *V*_*T*_ [ml/cm^3^], respectively. B) Myelin system–MR-based absolute myelin water content modality ($WC_{my}^{abs}$, [unitless]) is used as myelin density marker. C) Dopamine system–[^11^C]Raclopride PET imaging is used as D_2_/D_3_ receptor maker; [^123^I]FP-CIT SPET imaging is used as dopamine transporter (DAT) marker; [^18^F]FDOPA PET imaging refers to dopamine synthesis capacity (DDC, dopa decarboxylase enzymatic rate). The parametric maps are images of non-displaceable specific binding, *BP*_*ND*_ [unitless], index of uptake *IU* [unitless] and rate of net uptake, *K*_*i*_ [ml/cm^3^], respectively.

Finally we used MR-based absolute myelin water data available online (http://www.rheinahrcampus.de/Downloads.4916.0.html) as the image-based measure of myelin sheet density [14–16]. All image datasets were either freely available online or already previously published by the research group and can be found at the figshare repository (https://figshare.com/articles/Serotonin_and_dopamine_D2_PET_images/2068044; https://figshare.com/articles/MR_based_absolute_myelin_water_image/2068047; https://figshare.com/articles/Image_data/2068011). Details on acquisition processing and quantification procedures for each dataset as well as details on ethical approval granted by the local ethics committees are reported in the referenced literature. Fig 3 reports the templates used for the analysis.

The imaging data have been tested with matched and mismatched genomic information, i.e. the imaging templates were compared with mRNA data that were both related and non-related to their protein targets. Details on the target systems for each imaging modality and the tested genes are reported in Table 1.

**Table 1:** MENGA applications in representative imaging modalities.

*Image* Modality	Target system	Target function	Tested Gene	*Image* AUTO-correlation			*Gene* AUTO-correlation			*Univariate* CROSS-correlation				*Multivariate* CROSS-correlation		
				mean	±	SD	mean	±	SD	mean	±	SD	Slope	R_adj_^2^	Chance likelihood	Slope
***Matched cases***																
[^11^C]CUMI	Serotonin	5HT1A receptors	HTR1A	0.828	±	0.059	0.9	±	0.053	0.569	±	0.106	6/6	0.634	0%	(+)
[^11^C]WAY100635	Serotonin	5HT1A receptors	HTR1A	0.892	±	0.048	0.9	±	0.053	0.68	±	0.107	6/6	0.749	0%	(+)
MWC	Myelin	Myelin density	MBP	0.664	±	0.123	0.838	±	0.117	0.458	±	0.175	6/6	0.589	1%	(+)
			MOG	0.664	±	0.123	0.756	±	0.174	0.438	±	0.194	6/6	0.479	2%	(+)
			MOBP	0.664	±	0.123	0.773	±	0.16	0.384	±	0.179	6/6	0.646	0%	(+)
[^11^C]Raclopride	Dopamine	D2 receptors	DRD2	0.662	±	0.249	0.897	±	0.036	0.323	±	0.169	6/6	0.421	1%	(+)
[^123^I]FT-CIT	Dopamine	Dopamine transporter	SLC6A3	0.787	±	0.15	0.269	±	0.224	0.193	±	0.157	6/6	0.05	2%	(+)
[^18^F]FDOPA	Dopamine	Dopamine synthesis	DDC	0.743	±	0.173	0.793	±	0.136	0.32	±	0.156	6/6	0.22	4%	(+)
***Mismatched cases***																
[^11^C]CUMI	Serotonin	5HT1A receptors	DRD2	0.828	±	0.059	0.897	±	0.036	0.161	±	0.128	6/6	0.030*	16%	(-)
[^123^I]FT-CIT	Dopamine	Dopamine synthesis	HTR1A	0.787	±	0.15	0.9	±	0.053	n.a.	±	n.a.	n.a.	0.442*	12%	(-)
MWC	Myelin	Myelin density	DDC	0.664	±	0.123	0.793	±	0.136	0.146	±	0.104	6/6	0.194*	25%	(+)

All the correlation estimates refer to Pearson’s correlation coefficient R^2^ (auto-correlation and univariate cross-correlation) or the adjusted R-squared coefficient of determination R_adj_^2^ (multivariate cross-correlation). Mean±SD refer to average and standard deviation across the 6 donors of the Allen Brain atlas. Slope: it returns information about the directionality of the correlation; for the univariate correlation, it is the number of times (out of 6) that there is direct (positive) correlation, for the multivariate correlation, it represent the direction of the correlation respect to the first principal component (+/-)

Gene list: HTR1A, serotonin 5HT1A receptor; MBP: myelin basic protein; MOG: myelin oligodendrocyte glycoprotein; MOBP: myelin-associated oligodendrocytic basic protein; DRD2: dopamine D2 receptor; SLC6A3: dopamine active transporter; DDC: DOPA decarboxylase.

* indicates negative correlations

All the analysis were carried out with the same default settings, by using the simplified coarse region list, the default MNI mask (limited to the left hemisphere) and a window size of 5 mm.

### Software applications in imaging-genomic matched cases

The results of MENGA analysis in the matched studies are reported in Table 1. Image auto-correlation of PET-derived templates of the serotonin system was the highest among the tested cases. The auto-correlation for the antagonist [^11^C]WAY100635 was somewhat higher than the one of the agonist [^11^C]CUMI (on average R^2^ = 0.892±0.048 and R^2^ = 0.828±0.059 respectively). On the contrary, the myelin water content template showed the smallest image auto-correlation (R^2^ = 0.664±0.123). This indicates limited spatial homogeneity, since a slight difference in the ABA coordinates of the genomic sampling across the donors leads to different image sample values that are then reflected by a small auto-correlation.

**Fig 4:**
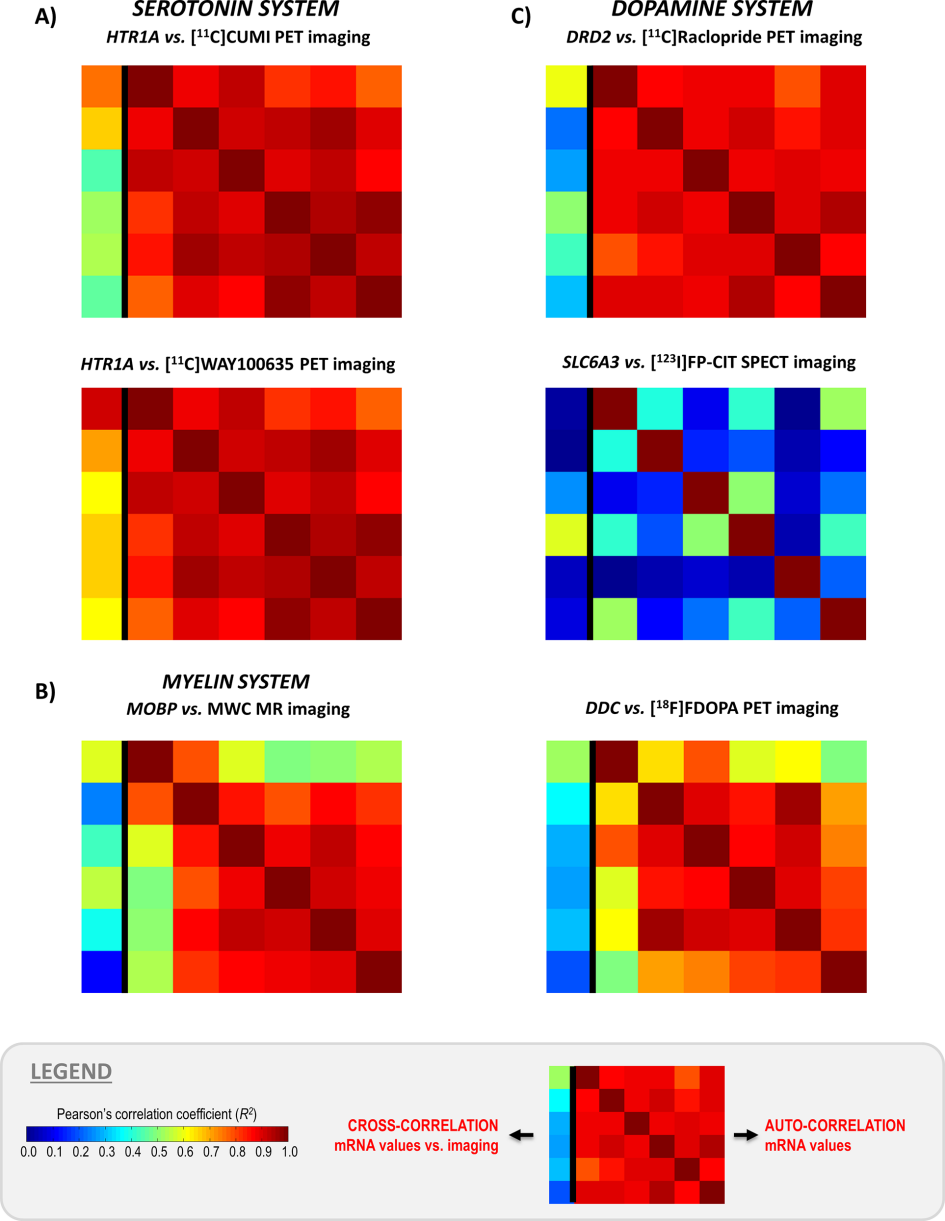
MENGA applications in representative imaging modalities with matched genomic data. The figure reports the genomic-imaging cross-correlation values along with genomic auto-correlation. In the results matrix, each element represents the correlation between couples of donors (for the auto-correlation) or between one donor’s mRNA levels and the image sampled in the same donor’s space (for the cross-correlation). A) Serotonin system: [^11^C]CUMI and [^11^C]WAY100635 PET imaging vs. 5HT_1A_ receptor (HTR1A) mRNA expression; B) Myelin system: myelin water content MR imaging vs. myelin-associated oligodendrocyte basic protein (MOBP) mRNA expression C) Dopamine system: [^11^C]Raclopride PET imaging vs. dopamine D_2_ receptor (DRD2) mRNA expression; [^123^I]FP-CIT SPET imaging vs dopamine transporter (DAT) mRNA expression; [^18^F]FDOPA PET imaging vs. dopa decarboxylase (DDC) mRNA expression.

The genomic auto-correlation and the genomic-imaging cross-correlation values are reported in Fig 4. In the results matrix, each element represents the correlation between couples of donors (for the auto-correlation, 6x6 values) or between one donor’s mRNA levels and the image sampled in the same donor’s space (for the cross-correlation, 6x1 values). This way of representing of the results is useful to capture at first glance the more consistent gene systems. From Fig 4 it is clear that the between donors correlation values of mRNA data were high for all tested systems (average values R^2^ > 0.75) with the only exception of the dopamine transporter (on average R^2^ = 0.269±0.224, Table 1). The lower correlation for DAT was expected since functionally modulated proteins are characterized by higher biological variability of the mRNA data. In fact DAT expression is modulated by endogenous dopamine and it is expected to be highly variable across donors [17]. On the contrary, dopamine synthesis capacity does not vary across donors and its mRNA expression is characterized by high auto-correlation, despite depending from an enzyme and not a structural protein [18].

**Fig 5:**
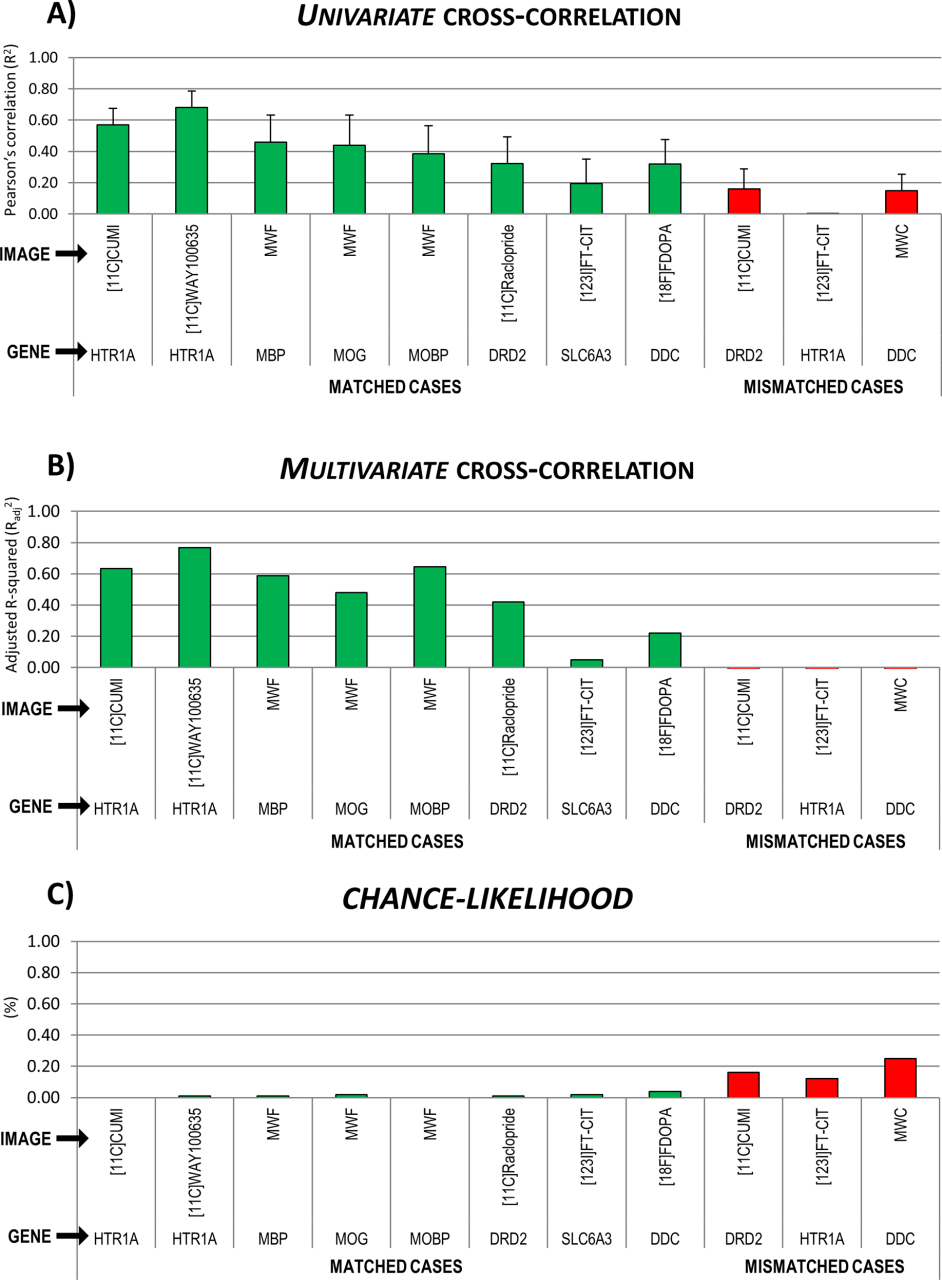
Summary statistics of MENGA applications in representative imaging modalities with matched and mismatched genomic data. Cross-correlation results as A) univariate Pearson’s R^2^, B) multivariate correlation coefficient and C) chance-likelihood associated to the R-squared are reported for all the sets of image vs. genomic applications, both for matched (green bars) and mismatched (red bars) cases. Univariate correlations are presented as mean and variability across donors.

Univariate and multivariate cross-correlations are reported in Table 1 and Fig 5. Univariate cross-correlation with mRNA data for the serotonin PET templates were the highest among the tested case and also showed the smallest between donors variability (R^2^ = 0.680±0.107 for [^11^C]WAY100635 and R^2^ = 0.569±0.107 for [^11^C]CUMI). Myelin and dopamine data showed comparable univariate cross-correlations between imaging and mRNA templates (R^2^ between 0.320±0.156 and 0.458±0.175) but lower for the SPET-DAT (R^2^ = 0.193±0.157).

Multivariate cross-correlation values showed the same trend as univariate results, but the coefficient of determination values obtained was generally higher (Table 1, Fig 5). These were estimated with high reliability, with values of chance likelihood smaller than 2% for the serotonin and myelin and smaller than 5% for the dopamine pathways.

### Software applications in imaging-genomic mismatched cases

In addition to comparing imaging data with the corresponding target mRNA expression levels, we also applied MENGA to a series of mismatched image-genomic cases. Three image templates were correlated to mRNA data of unrelated biological system. This was done to exclude the possibility that MENGA returns correlation values induced by spurious confounding factors.

We tested the serotonin [^11^C]CUMI template with the dopamine D_2_ receptor gene, the dopamine SPET data with the 5HT_1A_ serotonin receptor gene and the myelin template with the dopamine synthesis capacity mRNA. Results for the mismatched analysis are reported in Table 1 and Fig 5.

**Fig 6:**
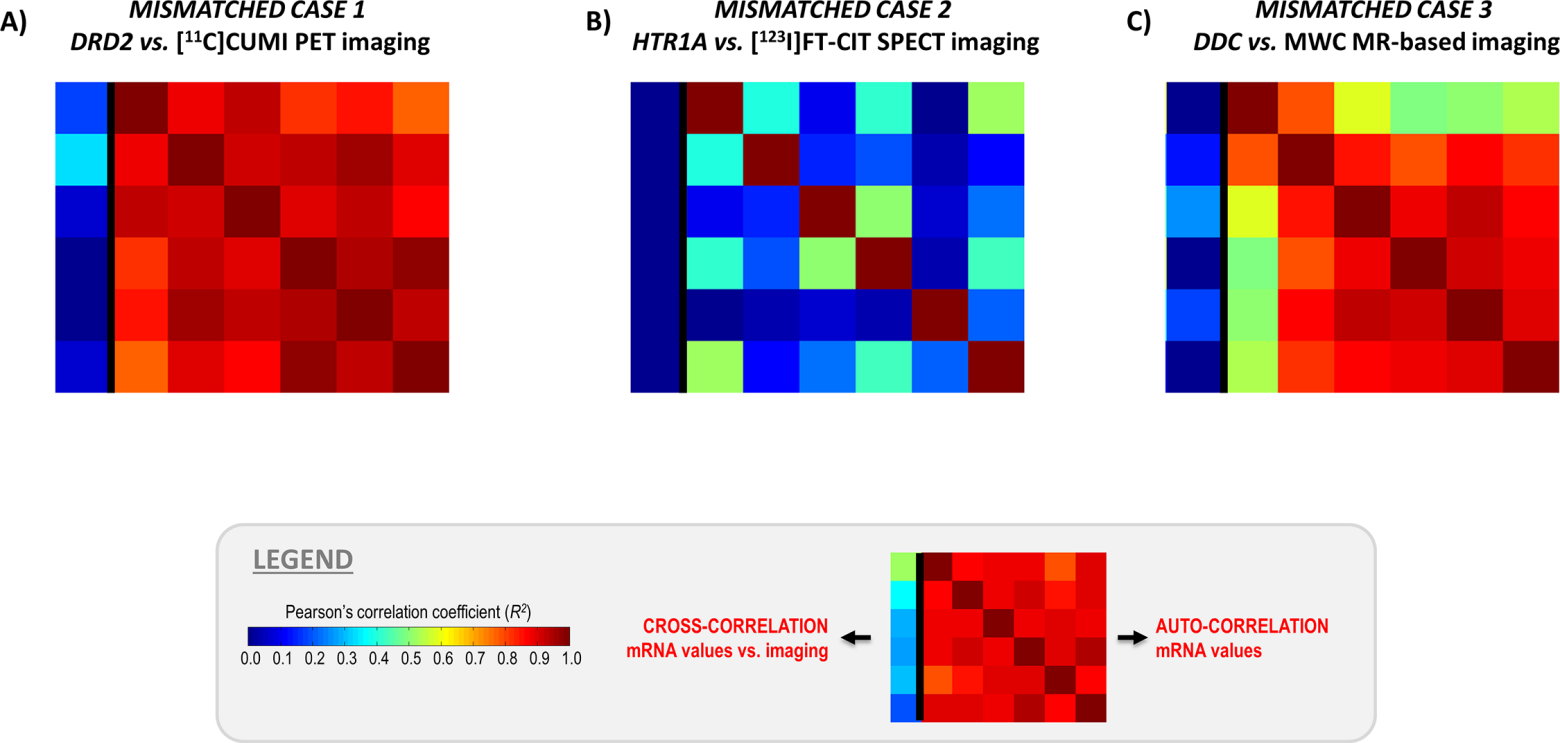
MENGA applications in representative imaging modalities with mismatched genomic data. The figure reports the genomic-imaging cross-correlation values along with genomic auto-correlation. In the results matrix, each element represents the correlation between couples of donors (for the auto-correlation) or between one donor’s mRNA levels and the image sampled in the same donor’s space (for the cross-correlation) in three different cases of images vs. mismatched genomic data integration. A) [^11^C]CUMI PET imaging vs. D_2_ receptor (DRD2) mRNA expression; B) [^123^I]FP-CIT SPET imaging vs 5HT_1A_ receptor (HTR1A) mRNA expression; C) myelin water content MR imaging vs. dopa decarboxylase (DDC) mRNA expression.

Both univariate and multivariate cross-correlation values were negligible and significantly lower than those obtained in the matched cases (R^2^ < 0.2, when available, Table 1). This can also be seen directly from the genomic auto-correlation and the genomic-imaging cross-correlation matrixes reported in Fig 6.

Moreover, the chance likelihood of the multivariate correlation was high (from 12% for the [^123^I]FP-CIT-5HT_1A_ match to 25% for the myelin water content-DDC match), indicating a high probability for $R_{adj}^{2}$ to be due to chance (Table 1, Fig 5).

## Discussion

In this work we presented MENGA, an integrated system for the comparison of genomic and imaging data. The software probes the relationship between an image end-point and a pre-selected list of genes of the ABA database, by quantitative evaluation of spatial mRNA expression patterns and image data.

### Implementative choices and statistics

MENGA has been created to enable the exploitation of the full potential of ABA database for imaging-genomic integration by the scientific community. MENGA has been built as a user friendly tool and is usable by people with limited programming computer knowledge.

For this reason, the choice of settings for the analysis are kept to a minimum and the choice of input/output format files was made to maximize the usability of the program. The management of.txt files can be performed by most of computer applications and the Analyze/NIfTI files represent the current standard for medical imaging.

Still the most critical aspect of MENGA usage may be the settings definition. To this end a set of pre-compiled libraries is offered to the user as default setting for both the list of regions and the mask. The experienced user can however extend this set with additional solutions depending on his/her needs. The main choice regarding the mask is whether to use the MNI whole brain mask or limit the analysis to the left hemisphere. We set the MNI mask of the left hemisphere as default in order to perform the analysis consistently in the same brain area for all the donors to avoid introducing artifacts in the results due to the possible asymmetry of the biological system under study.

Another key setting is represented by the window size used to resample the image data into each donor’s space. This value must be based on the resolution of the imaging modality analyzed with MENGA. However, one has to consider the noise of the image modality and the impact that a small window size might have in the resampling, even for high resolution images such as anatomical MRI. The default value is 5 mm to match imaging data acquired at discrete spatial resolution such as PET or other smoothed image modalities. The impact of this setting is mainly on the number of samples which are then included in the analysis: for increasing value of the window size the number of samples eliminated will also increase because there will be more elements out of the MNI mask (up to 18% of the total number of samples on average for a window size of 11 mm), see Appendix for details.

### Performance on the representative tested cases

The results obtained by MENGA on the paradigmatic set of images were consistent with our *a priori* hypotheses. The highest cross-correlation was found for the serotonin system (the receptor 5HT1A) where no post-translational modifications have been reported [19]. For this system, mRNA profiles can predict 57–77% of the spatial variability of the receptor concentration in the brain. We found reduced cross-correlation between mRNA activity and the agonist ligand [^11^C]CUMI compared to the antagonist [^11^C]WAY100635 for the same site, confirming our previous results [2].

Cross-correlation results for myelin and dopamine systems were comparable with the only exception of DAT data that also presented the smallest genomic auto-correlation. This may be expected as mRNA levels are better predictors of concentrations for structural than functional proteins [2,6].

The lack of correlation found in the integration of imaging data and mismatched information eliminates the possibility that the similarities found in the spatial patterns are due to spurious correlations. The image auto-correlation analysis among the modalities highlights the different impact of the image re-sampling in donors’ space.

### Probe selection

In ABA database, only 30% of the genes are represented by a single probe, 51% of the genes are described by two probes and 19% of the genes by three or more. The presence of multiple probes is not necessarily an advantage, as ABA includes the probes with varying specificity or sensitivity to the target. The presence of probes with very inconsistent expression profiles for the same gene may prevent the possibility of simply considering the mean mRNA expression as representative of the true mean transcript expression. It follows that it is fundamental for any type of application to select only the probe that best describes the real transcript profile and discard the remaining ones.

In this work we implemented a data-driven method for the probe selection. The approach accounts for between-donor consistency and probe information content to retain the representative expression profile for a given gene, which in turn is used for MENGA integration analysis; please refer to the Appendix for a detailed discussion. Note that the processed version of ABA database included in MENGA also contains all the probe profiles in log2 intensity prior the normalization in z-score, so that the expert user has the freedom to apply the software to the raw data implementing his/her criteria of choice for the probe selection.

### Results interpretation

MENGA returns the imaging-genomic cross-correlation values to give a quantitative assessment of the amount of the variability in the image phenotype that can be explained by spatial mRNA expression patterns. High values of cross-correlation indicate a good correspondence of image data and mRNA information. In presence of low cross-correlation values, it is necessary to distinguish between univariate and multivariate results. In particular, low average values of univariate cross-correlations (especially with high between donors standard deviation) can indicate high variability in the biological gene process under study, which is likely to also lead to low genomic auto-correlation estimates.

In this case the interpretation of multivariate results must be considered with care due to the presence of the PCA step in the analysis. The presence of the PCA step is not a problem in the presence of high genomic auto-correlation. In this case, the process is characterized by low variability and all the donors carry similar mRNA information and PCA will return few components (even one component could be enough to account for 95% of the data variability) all similarly correlated with the image data with no risk of overfitting. On the other hand, in the presence of a highly variable biological process, a larger number of highly non collinear principal components will be required to describe the genomic data. As a consequence, the multivariate correlation could be only driven by the component the most correlated with the image and overestimate the true correlation present in the data.

## Conclusions

MENGA is an integrated system that allows the comparison of genomic and imaging data. The software uses mRNA brain maps of the whole human genome derived from the Allen human brain atlas and it has can be used in conjunction with any imaging modality. Specifically, it explores the similarities between the spatial patterns of genomic expressions of a pre-selected list of genes and brain imaging data of unrelated subjects.

## Appendix

### ABA database processing

The original ABA dataset consisted in the complete microarray datasets for the full complement of six brains that were downloaded from http://human.brain-map.org/static/download. The datasets contained the probe expression values normalized across all brains using the normalization process described in the technical white paper, *Microarray Data Normalization* presented in the same website. For each donor, ABA makes available all normalized microarray samples for all probes in log2 value as well as probe and sample metadata necessary for the processing analysis.

The dataset is derived from 6 healthy donors (5 males, 1 female, age: 42.5 ± 13.4, range 24–57 yrs) and contains 29’177 gene expression profiles sampled throughout the brain. The number of probes was consistent between genes (on average 2.0 ± 1.4 probes per gene). In particular, 51% of the genes had 2 probes, 30% and 13% of genes had 1 and 3 probes respectively and only 6% of genes had more than 3 probes. On average, 617 ± 242 samples were collected for each donor to represent all the brain structures proportionally to their volume. For two out of the six brains, samples were collected from both hemispheres. For the remaining four, tissue samples for microarray analysis were limited to the left hemisphere. Full details about the procedures for the tissue collection and processing, the microarray experimental design and execution, and the data quality control up to the integration of the data into the online resource are reported in the supplementary data of [1].

The first step of our processing of ABA database consisted in the organization of the dataset in gene folders each containing all the probes for a given gene. For each donor, samples data for all probes were converted in z-score (using mean and standard deviation as normalization factors within a given subject) which were then used for all the analyses. Note that the different number of samples per donor causes negligible impact on the z-score calculation because the number of samples is such to allow a valid normalization (more than 400 samples per donor). Finally, we defined a unique mRNA profile for each gene by selecting the probe that best the gene expression profile, evaluating the data from all donors at the same time. One probe was retained as representative of the particular gene transcript and it was the same for all donors. We chose to select the probe on the basis of the distribution of the expression values in z-score for all donors. In this approach data from all donors are combined together creating a unique vector on which the distribution analysis is performed: the probe with the most symmetric and least skewed distribution is chosen as representative of the gene profile. Specifically, we created the histogram of the distribution for each probe and then we located its maximum: we selected the one with the peak located utmost on the right or, if all the probes presented the maximum in the same bin, we chose the probe with the lowest peak. This approach is different than the one presented in [1], where the probe with the highest between donors auto-correlation was considered as representative of the particular gene transcript.

Since the number of probes varies across the genes, we considered two different cases:

1. one probe: the probe is necessarily selected;
2. two or more probes: we evaluated the distribution of the expression values and chose the one with the most symmetric and least skewed profile to avoid the non-linearity effect on the microarray measures.

The probe selected was then selected as unique gene profile and stored in the processed version of ABA database. This is available in MENGA distribution package.

### Performance statistics of pre-processing analysis

We assessed the impact of using different window sizes on the percentage of pairs of samples that would be included in the same image sample (*overlapping samples* with a certain window size) and on the percentage of image samples eliminated when using the MNI mask (*boundary samples*). The minimum distance between two different genomic samples was 1 mm (i.e. there were adjacent samples) but these represented less than 0.1% of the total number of samples on average across the donors (mean ± SD: 0.03% ± 0.02%) (S2 Table). Even when considering a window of 11 x 11 x 11 elements, the overlapping samples counted for less than 2% of total samples.

The size of the window had a greater impact on the percentage of voxels eliminated when using the MNI whole brain mask. This percentage varied across donors as consequence of the different sampling of ABA (in the whole brain for the first two donors and only in the left hemisphere for the other four donors) (S3 Table). In general, the greater the window size, the higher the percentage of voxels discarded.

We also evaluated the number of samples available for each region of the simplified coarse list in the best scenario of having all the samples available (i.e. MNI whole brain mask) and a representative set of regions. The number of samples was consistent across donors and each regions was well represented (between donors mean and SD: 35 ± 27 samples per region) (S4 Table). To note that the first two donors (both hemispheres analyzed) had twice the number of samples of the remaining four donors (only with the left hemisphere).

## Supporting Information

S1 Table List of regions of the coarse and simplified coarse level. The ABA labels and the corresponding names of the ROIs for the coarse level and the simplified coarse level of resolution included in MENGA are reported.
(DOCX)

S2 Table Summary statistics of overlapping samples. The percentage of pairs of samples that would be included in the same window (*overlapping samples*) for a certain window size is reported for various size values (from 1 to 11 mm) for each donor. The mean, standard deviation, minimum and maximum across donors are also reported.
(DOCX)

S3 Table Summary statistics of boundary samples. The percentage of samples eliminated because including NaN elements, i.e. elements outside the MNI whole brain mask (*boundary samples*), is reported for various size values (from 1 to 11 mm) for each donor. The mean, standard deviation, minimum and maximum across donors are also reported.
(DOCX)

S4 Table Summary statistics of number of ABA samples. The number of samples included in the regions listed (*simplified coarse list of ROIs*) is reported for each donor. The mean, standard deviation, minimum and maximum across regions are also reported. The MNI whole brain mask was used to include all the available samples. In fact the number of samples for the first two donors (with both hemispheres analyzed) is at least twice the number of samples of the four remaining donor.
(DOCX)
