# Supplementary material for: MENGA: A New Comprehensive Tool for the Integration of Neuroimaging Data and the Allen Human Brain Transcriptome Atlas
Source: PLoS One. 2016 Feb 16;11(2):e0148744. doi: 10.1371/journal.pone.0148744 (PMC4755531; doi:10.1371/journal.pone.0148744)
Supplement: S1 Table — The ABA labels and the corresponding names of the ROIs for the coarse level and the simplified coarse level of resolution included in MENGA are reported. (DOCX) [file pone.0148744.s001.docx]

**Table S1. List of** **regions of the coarse and simplified coarse level.**

| **Coarse level** | | **Simplified coarse level** | |
| --- | --- | --- | --- |
| **ABA label** | **Regions name** | **ABA label** | **Regions name** |
| 4005 | brain | 4009 | frontal lobe |
| 4009 | frontal lobe | 4084 | parietal lobe |
| 4084 | parietal lobe | 4132 | temporal lobe |
| 4132 | temporal lobe | 4180 | occipital lobe |
| 4180 | occipital lobe | 4220 | cingulate gyrus |
| 4220 | cingulate gyrus | 4242, 4249 | hippocampus |
| 4242 | parahippocampal gyrus | 4268 | insula |
| 4249 | hippocampal formation | 4277 | striatum |
| 4268 | insula | 4293 | globus pallidus |
| 4277 | striatum | 4300 | basal forebrain |
| 4293 | globus pallidus | 4321 | claustrum |
| 4300 | basal forebrain | 4327 | amygdala |
| 4321 | claustrum | 4393, 4504, 4517, 4540 | thalamus |
| 4327 | amygdala | 4697, 4780 | cerebellum |
| 4393 | dorsal thalamus | 9001, 9132, 9135, 9512 | brainstem |
| 4504 | ventral thalamus |  |  |
| 4517 | subthalamus |  |  |
| 4520 | epithalamus |  |  |
| 4540 | hypothalamus |  |  |
| 4697 | cerebellar cortex |  |  |
| 4780 | cerebellar nuclei |  |  |
| 9001 | mesencephalon |  |  |
| 9132 | basal part of pons |  |  |
| 9135 | pontine tegmentum |  |  |
| 9218 | white matter |  |  |
| 9352 | sulci & spaces |  |  |

The ABA labels and the corresponding names of the ROIs for the coarse level and the simplified coarse level of resolution included in MENGA are reported.
