# Supplementary material for: MENGA: A New Comprehensive Tool for the Integration of Neuroimaging Data and the Allen Human Brain Transcriptome Atlas
Source: PLoS One. 2016 Feb 16;11(2):e0148744. doi: 10.1371/journal.pone.0148744 (PMC4755531; doi:10.1371/journal.pone.0148744)
Supplement: S2 Table — The percentage of pairs of samples that would be included in the same window (overlapping samples) for a certain window size is reported for various size values (from 1 to 11 mm) for each donor. The mean, standard deviation, minimum and maximum across donors are also reported. (DOCX) [file pone.0148744.s002.docx]

**S2** **Table. Summary statistics of overlapping samples.**

| **% overlapping samples** | **1 mm** | **3 mm** | **5 mm** | **7 mm** | **9 mm** | **11 mm** |
| --- | --- | --- | --- | --- | --- | --- |
| Donor 09861 | 0.02 | 0.07 | 0.20 | 0.43 | 0.73 | 1.11 |
| Donor 10021 | 0.01 | 0.07 | 0.23 | 0.49 | 0.88 | 1.34 |
| Donor 12876 | 0.06 | 0.18 | 0.37 | 0.56 | 0.85 | 1.11 |
| Donor 14380 | 0.04 | 0.11 | 0.30 | 0.56 | 0.90 | 1.35 |
| Donor 15496 | 0.05 | 0.21 | 0.47 | 0.89 | 1.37 | 1.90 |
| Donor 15697 | 0.03 | 0.11 | 0.28 | 0.50 | 0.75 | 1.16 |
| **mean** | **0.03** | **0.13** | **0.31** | **0.57** | **0.91** | **1.33** |
| **STD** | **0.02** | **0.06** | **0.10** | **0.16** | **0.23** | **0.30** |
| **min** | **0.01** | **0.07** | **0.20** | **0.43** | **0.73** | **1.11** |
| **max** | **0.06** | **0.21** | **0.47** | **0.89** | **1.37** | **1.90** |

The percentage of pairs of samples that would be included in the same window (*overlapping samples*) for a certain window size is reported for various size values (from 1 to 11 mm) for each donor. The mean, standard deviation, minimum and maximum across donors are also reported.
