# Supplementary material for: MENGA: A New Comprehensive Tool for the Integration of Neuroimaging Data and the Allen Human Brain Transcriptome Atlas
Source: PLoS One. 2016 Feb 16;11(2):e0148744. doi: 10.1371/journal.pone.0148744 (PMC4755531; doi:10.1371/journal.pone.0148744)
Supplement: S3 Table — The percentage of samples eliminated because including NaN elements, i.e. elements outside the MNI whole brain mask (boundary samples), is reported for various size values (from 1 to 11 mm) for each donor. The mean, standard deviation, minimum and maximum across donors are also reported. (DOCX) [file pone.0148744.s003.docx]

**S3 Table. Summary statistics of boundary samples.**

| **% boundary samples** | **1 mm** | **3 mm** | **5 mm** | **7 mm** | **9 mm** | **11 mm** |
| --- | --- | --- | --- | --- | --- | --- |
| Donor 09861 | 0.00 | 0.00 | 1.59 | 2.96 | 6.13 | 10.04 |
| Donor 10021 | 0.22 | 1.12 | 2.35 | 4.26 | 6.72 | 11.42 |
| Donor 12876 | 1.10 | 5.23 | 8.54 | 11.29 | 15.15 | 18.18 |
| Donor 14380 | 1.32 | 6.05 | 9.83 | 12.29 | 15.31 | 19.47 |
| Donor 15496 | 4.04 | 5.32 | 7.87 | 10.43 | 14.26 | 23.19 |
| Donor 15697 | 3.79 | 6.99 | 10.58 | 14.17 | 17.37 | 21.56 |
| **mean** | **1.75** | **4.12** | **6.79** | **9.23** | **12.49** | **17.31** |
| **STD** | **1.76** | **2.85** | **3.86** | **4.55** | **4.81** | **5.40** |
| **min** | **0.00** | **0.00** | **1.59** | **2.96** | **6.13** | **10.04** |
| **max** | **4.04** | **6.99** | **10.58** | **14.17** | **17.37** | **23.19** |

The percentage of samples eliminated because including NaN elements, i.e. elements outside the MNI whole brain mask (*boundary samples*), is reported for various size values (from 1 to 11 mm) for each donor. The mean, standard deviation, minimum and maximum across donors are also reported.
