# Supplementary material for: MENGA: A New Comprehensive Tool for the Integration of Neuroimaging Data and the Allen Human Brain Transcriptome Atlas
Source: PLoS One. 2016 Feb 16;11(2):e0148744. doi: 10.1371/journal.pone.0148744 (PMC4755531; doi:10.1371/journal.pone.0148744)
Supplement: S4 Table — The number of samples included in the regions listed (simplified coarse list of ROIs) is reported for each donor. The mean, standard deviation, minimum and maximum across regions are also reported. The MNI whole brain mask was used to include all the available samples. In fact the number of samples for the first two donors (with both hemispheres analyzed) is at least twice the number of samples of the four remaining donor. (DOCX) [file pone.0148744.s004.docx]

**S4 Table. Summary statistics of number of ABA samples.**

| **ROI** | **Donor 9861** | **Donor 10021** | **Donor 12876** | **Donor 14380** | **Donor 15496** | **Donor 15697** | **MEAN** | **SD** |
| --- | --- | --- | --- | --- | --- | --- | --- | --- |
| Frontal Lobe | 149 | 123 | 27 | 36 | 41 | 38 | **69.0** | **52.8** |
| Parietal Lobe | 62 | 41 | 29 | 26 | 25 | 25 | **34.7** | **14.7** |
| Temporal Lobe | 145 | 77 | 26 | 45 | 30 | 40 | **60.5** | **45.2** |
| Occipital Lobe | 31 | 36 | 21 | 30 | 30 | 25 | **28.8** | **5.2** |
| Cingulate Gyrus | 21 | 31 | 25 | 24 | 25 | 22 | **24.7** | **3.5** |
| Hippocampus | 76 | 63 | 19 | 31 | 25 | 27 | **40.2** | **23.4** |
| Insula | 12 | 7 | 4 | 8 | 7 | 6 | **7.3** | **2.7** |
| Striatum | 48 | 46 | 16 | 24 | 18 | 18 | **28.3** | **14.7** |
| Globus Pallidus | 11 | 14 | 4 | 2 | 3 | 5 | **6.5** | **4.8** |
| Basal Forebrain | 7 | 10 | 7 | 9 | 6 | 12 | **8.5** | **2.3** |
| Claustrum | 17 | 11 | 5 | 7 | 5 | 2 | **7.8** | **5.4** |
| Amygdala | 14 | 22 | 8 | 6 | 7 | 9 | **11.0** | **6.1** |
| Thalamus | 66 | 84 | 45 | 50 | 38 | 42 | **54.2** | **17.6** |
| Cerebellum | 51 | 75 | 30 | 27 | 43 | 46 | **45.3** | **17.3** |
| Brainstem | 160 | 190 | 27 | 84 | 81 | 66 | **101.3** | **61.3** |
|  |  |  |  |  |  |  |  |  |
| **mean** | **58.0** | **55.3** | **19.5** | **27.3** | **25.6** | **25.5** | **35.2** | **18.5** |
| **SD** | **53.1** | **49.9** | **12.0** | **21.3** | **20.5** | **18.1** | **27.4** | **19.3** |
| **min** | **7** | **7** | **4** | **2** | **3** | **2** | **6.5** | **2.3** |
| **max** | **160** | **190** | **45** | **84** | **81** | **66** | **101.3** | **61.3** |

The number of samples included in the regions listed (*simplified coarse list of ROIs*) is reported for each donor. The mean, standard deviation, minimum and maximum across regions are also reported. The MNI whole brain mask was used to include all the available samples. In fact the number of samples for the first two donors (with both hemispheres analyzed) is at least twice the number of samples of the four remaining donor.
